# Peer Support for Individuals with Major Limb Loss: a Scoping Review

**DOI:** 10.33137/cpoj.v6i1.42170

**Published:** 2023-12-28

**Authors:** A Costa-Parke, A.M Di Lella, A Walker, L Verweel, C MacKay

**Affiliations:** 1 Department of Occupational Science and Occupational Therapy, University of Toronto, Toronto, Canada.; 2 West Park Healthcare Centre, Toronto, Canada.; 3 Department of Physical Therapy, University of Toronto, Toronto, Canada.

**Keywords:** Amputation, Peer Support, Limb Loss, Scoping Review, Social Support, Prosthesis, Rehabilitation

## Abstract

**BACKGROUND:**

Major limb loss can have profound physical and psychosocial implications for individuals, impacting their quality of life and well-being. Despite the effectiveness of peer support in improving outcomes for various chronic conditions, its impact on individuals with major limb loss remains understudied.

**OBJECTIVE(S):**

This review aims to explore the existing literature on peer support for individuals with major limb loss. Specifically, exploring how the literature defines peer support; examining its implementation, identifying outcomes measured in peer support interventions, assessing the benefits for individuals with major limb loss, and identifying barriers associated with peer support provision.

**STUDY DESIGN:**

This review followed Arksey and O'Malley's methodological framework, analysing relevant literature to identify evidence, definitions, and key factors related to peer support for individuals with major limb loss.

**METHODOLOGY:**

A comprehensive search in January 2023 utilized databases: MEDLINE, PsychInfo, Embase, and CINAHL. After a two-phase screening process, articles meeting specific criteria were included. Thematic and descriptive numerical analyses were applied to the extracted data.

**FINDINGS:**

Twenty-two articles were reviewed. Peer support was described as an opportunity to provide education, advice, and encouragement between individuals with lived experiences. Across the two intervention-based studies investigating peer support programs, outcome measures included physical, psychological, social, and quality of life. Qualitative studies described perceived benefits as improved psychosocial well-being and the opportunity to exchange knowledge. Perceived barriers included a lack of formal training and male-dominated groups, which deterred individuals with amputation from participating.

**CONCLUSION:**

The evidence from the findings of the review sheds light on the current understanding of peer support for individuals with amputation. Due to the limited number of studies available, future research is necessary to develop and evaluate the effectiveness of peer support interventions tailored to this population.

## INTRODUCTION

Major limb loss is the partial or total amputation of an extremity that occurs at and/or above the ankle or wrist.^1^ This type of amputation may result from diabetes or other vascular conditions, a traumatic event, cancer, or a congenital condition.^2^ Living with major limb loss can have profound physical implications for the individual.^3^ Evidence has shown that people with major limb loss experience reduced physical function, mobility, and pain.^3^ While the consequences of amputation can lead to physical and mobility limitations, research has suggested that psychosocial and mental health outcomes are also significant barriers to effective recovery post-amputation. People with limb loss can experience grief, social isolation, loss of self-esteem,^3–6^ and higher rates of depression, anxiety, and body image disorders.^7^ Limb loss is also known to impact social interactions, community participation, and engagement in daily activities.^3,6^ These challenges can negatively impact an individual's quality of life and psychosocial well-being.^6^ Despite substantial evidence of the psychosocial impacts of amputation^8,9^ rehabilitation programs often focus on physical recovery with limited resources targeting psychosocial support.

Peer support is a non-medical intervention that offers a supportive relationship between individuals who have shared experiences with a condition.^10,11^ Peer support has been shown to be an important component in the care of individuals dealing with various health conditions, such as substance abuse^12^ and mental health illnesses.^12,13^ Fortuna et al.^13^ used PeerTECH, a peer support program supplemented by technological use, and found improvements in quality of life, self-management, and self-efficacy for managing health conditions, including mental illnesses such as schizophrenia and bipolar disorders, and comorbidities like diabetes and cardiovascular disease. Proudfoot et al.^14^ examined the inclusion of peer support in a psychoeducational program and found that the peer support programs led to greater adherence to treatment compared to the unsupported program. In addition, the peer support group led to decreases in stigmatization, a reduction in anxiety and depression.^14^ Similarly, individuals with diabetes reported that community-based peer support programs led by trained peers showed improvements in symptoms of depression, communication with healthcare practitioners, healthier lifestyle behaviours and increased self-efficacy.^15^

Although there is a breadth of evidence available about peer support among other populations with chronic conditions (e.g., diabetes, mental illnesses), less is known about the nature and scope of research on peer support for individuals living with major limb amputation. A scoping review on peer support for trauma survivors, which included individuals with traumatic amputations, found that peer support provided trauma survivors with socioemotional support as well as assistance in daily management and life navigation post-injury.^16^ However, studies of peer support following trauma may not be transferable to all individuals with limb loss, most of whom have amputations due to diabetes.^17^ To address this gap, the purpose of this scoping review was to examine the extent, nature, and scope of existing literature on peer support for people living with major limb loss to inform future research and practice.

## METHODOLOGY

A scoping review was performed to identify existing literature and clarify the definitions and key factors associated with this topic.^18^ The review followed Arksey and O'Malley's five-stage methodological framework, which outlines: (i) identifying the research questions; (ii) identifying relevant studies; (iii) study selection; (iv) charting the data; and (v) collating, summarizing, and reporting the results.^19^

### Identifying Research Question (Stage 1)

The research questions used to guide the review were: (1) how does the current literature define peer support? (2) how has peer support been implemented (e.g., programs, interventions, informal supports) and at what stage (e.g., acute care, rehab, community)? (3) what are the outcomes measured in peer support interventions? (4) what are the benefits of peer support for the quality of life of individuals with major amputations, and (5) what are the risks or barriers to the provision of peer support?

### Identifying Relevant Studies (Stage 2)

Literature searches were conducted in a period spanning January 2023 to April 2023 using the electronic databases of **MEDLINE**, **PsychInfo**, **Embase**, and **CINAHL**. The search strategy was customized to each database and used key terms that included “peer support”, “amputation”, and “major limb loss” (see **Appendix A**–**D** for search strategies). To supplement these searchers, a hand search of reference lists of retrieved articles was also conducted to scan for additional relevant studies. The four databases and hand-searched articles were uploaded to the software Covidence. A two-phase screening process was conducted; the first involved the elimination of articles based on title and abstract, and the second involved a full-text review. To establish inter-rater reliability, two authors (Di Lella A. M and Costa-Parke A) separately conducted each phase and then met to reach a consensus. Disagreements between reviewers were resolved by consensus or by the decision of a third reviewer (MacKay C).

### Study Selection (Stage 3)

The following inclusion criteria were used to guide the search and retrieve the articles: (1) published in the English language; (2) individuals with a major amputation, including trauma-related, surgical-related (cancer, infection, vascular, etc.), and congenital; (3) age range: 18 years and older; (4) studies should include some aspect of peer support, either formal or informal; (5) participants have either received peer support or provided peer support (with or without training); (6) articles should include primary data. Articles were excluded if: (a) they focused on minor limb amputations; (b) they focused on amputations in youth populations; (c) dissertations, study protocols, editorials, and conference proceedings.

### Charting the Data (Stage 4)

The data-charting form was developed using an Excel sheet by two authors (Di Lella A. M and Costa-Parke A) to maintain the consistency of the variables being extracted. The authors independently extracted the data and met to reach a consensus on the variables. The data extracted were summarized and inputted into tables that included: (i) study details (e.g., title, authors, year, country); (ii) population characteristics and eligibility (e.g., type of major limb amputation); (iii) study design/methodology (e.g., study objectives, types of measures used); (iv) peer support (e.g., definition, delivery method, practice setting); and (v) major findings (e.g., how studies define peer support, outcome measures, risks, and benefits).

### Collecting, Summarizing and Reporting the Results (Stage 5)

After charting information from included studies, we produced a narrative account of findings in two ways. First, quantitative analysis was conducted including a frequency analysis of the extent, nature and distribution of the studies included in the review. Second, the findings were organized thematically. We used a descriptive approach which involved allocating concepts or characteristics relevant to each research objective into overall categories. This included comparing information across studies, combining similar concepts, and summarizing ideas. Two authors (Di Lella A. M and Costa-Parke A) independently categorized data extracted from the articles to summarize the key ideas identified across articles and later met to reach a consensus on the findings. Other members of the research team were consulted to enhance validity and reduce biases in the results of the study.

## RESULTS

### Overview of study characteristics

**Figure 1:**
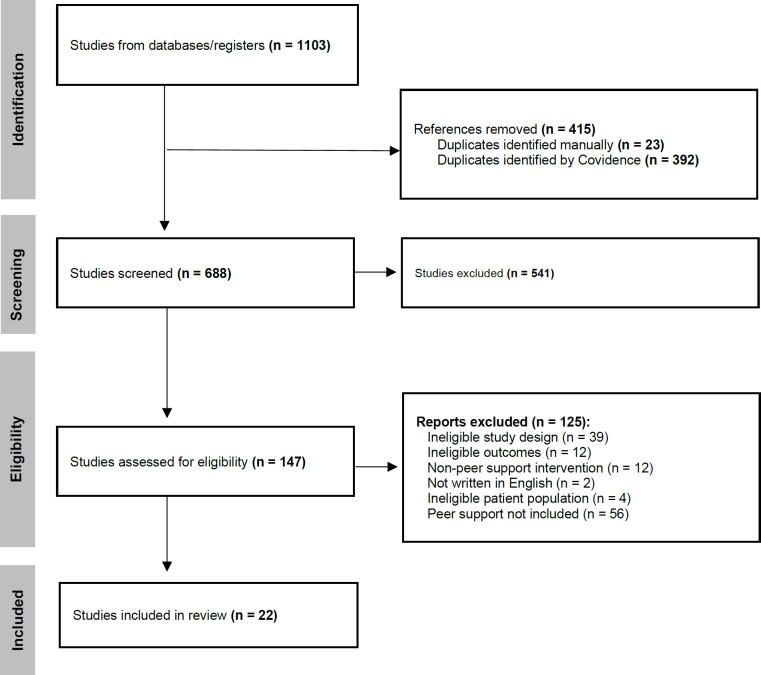
PRISMA diagram.

A total of 1103 articles were identified from four databases, and after duplicates were removed, 688 articles remained. The two authors (Di Lella A. M and Costa-Parke A) separately screened the titles and abstracts of the articles and a total of 147 were deemed eligible for full text review. Two authors (Di Lella A. M and Costa-Parke A) individually conducted a full-text review of these 147 and a total 22 articles met the inclusion criteria (**Figure 1**).

Articles reported primary data using qualitative methods (n=16), randomized controlled trials (RCT) (n=2), cross-sectional studies (n=2), cost-analysis (n=1), and non-controlled retrospective cohort study (n=1). Studies were conducted in Canada (n=4), the United States of America (n=7), Australia (n=4), the United Kingdom (n=2), the Democratic Republic of Congo (n=1), Scotland (n=1), Taiwan (n=1), and Iran (n=1). One of the studies by Anderson et al.^20^ was conducted in both the United Kingdom and Australia. The types of amputations discussed among the articles included participants with major lower extremity loss (n=15) or both major lower and upper extremity loss (n=7). Two studies reported on the effects of a peer support intervention. Seven articles explored the impacts of peer support as a primary objective, and 13 articles explored peer support as a secondary or other finding (i.e., the articles did not study peer support but generated findings that demonstrated peer support as relevant to its participants). See the full study details in **Table 1**.

**Table 1:** Study details.

Author (Year)	Location	Design	Study Objectives	Type of Amputation	Care Setting	Sample	Age Range (years)	Sex
Amorelli et al.^21^ (2019)	USA	Qualitative	To examine the components of the AU program through individual with amputation and healthcare professionals (HCPs) experiences, and to understand the value this program brings to the limb loss community.	Major upper and lower limb	Community	n = 10 (HCPs) n = 7 (amputees)	17+	n = 15 (male) n = 2 (female)
Andersen et al.^36^ (2023)	Democratic Republic of Congo	Non-controlled retrospective cohort study	To examine predictors of depression, anxiety, and stress prior to and following participation in the mental health and psychosocial support (MHPSS) intervention.	Major upper and lower limb	n/a	n = 132 (total) n = 100 (amputees)	0–70	n = 93 (male) n = 39 (female)
Anderson et al.^20^ (2022)	UK& Australia	Narrative exploration study (phenomenolo gical approach)	Examine the experiences and perspectives of prosthetic and orthotic users and explore their needs for the future.	Major lower limb	n/a	n = 5	33–67	n = 2 (male) n = 3 (female)
Anderson et al.^24^ (2019)	Australia	Qualitative (naturalistic enquiry)	To understand participant motivation to join a mobility clinic, examine their experiences, and understand its perceived benefits to their mobility.	Major lower limb	Community	n = 9	25–60	n = 3 (male) n = 6 (female)
Brusco et al.^27^ (2023)	Australia	Cost analysis	Explore the cost, impact, and willingness to pay for an Amputee Peer Support Program; a program offered by Limbs 4 Life.	n/a	Community	n = 38 (HCPs) n = 86 (program volunteers) n = 12 (program participants)	n (mean age): 40.7 (HCPs) 59.2 (program volunteers) 70.2 (program participants)	HCP n = 8 (male) n = 5 (female) Program volunteers n = 58 (male) n = 28 (female) Program participants n = 8 (male) n = 4 (female)
Dillon et al.^34^ (2020)	Australia	Qualitative (narrative inquiry approach)	To understand the experiences of individuals who have had sequential partial foot and transtibial amputations.	Major lower limb	Hospital & Community	n = 10	21–73	n = 8 (male) n = 2 (female)
Keeves et al.^38^ (2022)	Australia	Exploratory qualitative study	To determine the factors that facilitate and impede social and community participation following traumatic lower limb amputations.	Major lower limb	n/a	n = 9	50–64	n = 7 (male) n = 2 (female)
Lehavot et al.^37^ (2022)	USA	National qualitative study	To understand the perspectives of women veterans with lower limb loss regarding prosthetic devices and care.	Major lower limb	n/a	n = 30	40–80	n = 30 (female)
Liu et al.^31^ (2010)	Taiwan	Qualitative (phenomenolo gical approach)	To explore the perspectives of Taiwanese people who experienced a lower extremity amputation pre- and six months post-surgery.	Major lower limb	Rehabilitati on	n = 22	56–84	n = 15 (male) n = 7 (female)
MacBride et al.^25^ (1980)	Canada	Qualitative	To examine individual withamputation perspectives on group meetings and to understand the psychological impact of amputation and its influence on the success of amputee programs.	Major upper and lower limb	Rehabilitati on	n/a	64 (median)	3:1 (male: female ratio)
MacKay et al.^39^ (2022)	Canada	Qualitative	To examine the experiences of individuals in the community who have lower extremity dysvascular amputation	Major lower limb	n/a	n = 35	72–86	n = 23 (male) n = 12 (female)
Mayo et al.^40^ (2022)	Canada	Qualitative descriptive	To examine the mental health needs of individuals with lower limb amputations and understand their perspectives on using iCBT as a coping strategy postamputation.	Major lower limb	n/a	n = 10	43–77	n = 9 (male) n = 1 (female)
McGill et al.^41^ (2021)	UK	Qualitative	Explores the physical, psychological, and social experiences of veterans with limb loss and examines the factors that facilitate their independence.	Major upper and lower limb	n/a	n = 32	40–95	n = 30 (male) n = 2 (female)
Messinger et al.^32^ (2018)	USA	Cross-sectional (interpretive phenomenolog ical approach)	To explore the social experiences and recovery outcomes of amputees in the Military Advanced Training Centre at Walter Reed National Military Medical Centre.	Major lower limb	Rehabilitati on	n = 20	25–45	n = 19 (male) n = 1 (female)
Mortimer et al.^28^ (2002)	Scotland	Qualitative	Examine the experiences of amputees with (1) phantom limb pain, (2) perceptions of the current information provided about phantom pain, and (3) opinions about what information should be provided.	Major lower limb	Community	n = 31	30–74	n = 18 (male) n = 13 (female)
Nathan & Winkler^26^ (2019)	USA	Cross-sectional survey design	To explore the reasons a person with amputation will join, leave, or return to a peer support group as well as understand the role of technology-based support groups for amputees.	Major upper and lower limb	n/a	n = 54	20–82	n = 36 (male) n = 18 (female)
Radenovic et al.^33^ (2022)	Canada	Qualitative descriptive and discovery oriented approach	Understand the experiences of individuals with major lower limb loss and the factors that influence their reintegration into the community.	Major lower limb	Rehabilitati on	n = 9	51–82	n = 7 (male) n = 2 (female)
Richardson etal.^42^ (2020)	UK	Qualitative (interpretative phenomenolog ical analysis)	To explore the experiences and perceptions of peer mentors delivering peer support interventions to lower limb amputees.	Major lower limb	Community	n = 8	56–84	n = 3 (male) n = 5 (female)
Stutts et al.^35^ (2015)	USA	Qualitative (interpretative phenomenolog ical analysis)	To explore the coping strategies, perceived social support, participation in support groups, and experiences of acceptance and growth in women with amputations.	Major upper and lower	n/a	n = 30	23–81	n = 30 (female)
Turner et al.^29^ (2021)	USA	Multisite, 2-arm cluster RCT with masked outcome assessment	Examine the effectiveness of the VETPALS intervention on physical, psychological, and quality of life domains for individuals with lower limb loss and understand the feasibility of incorporating this program into a national health care program.	Major lower limb	Community	n = 147	Control = 64.12 (mean) Treatment = 64.89 (mean)	Control n = 76 (male) Treatment n = 68 (male)
Valizadeh et al.^23^ (2014)	Iran	Qualitative content analysis	Explores the experiences of lower limb amputees and examines the influence of support sources on their ability to adapt to their amputation.	Major lower limb	n/a	n = 20	25–57	n = 17 (male) n = 3 (female)
Wegener et al.^30^ (2009)	USA	RCT	Examined the effectiveness of a community-based self-management program on health outcomes for individuals with limb loss.	Major upper and lower limb	Community	n = 502	Control = 56.9 (mean) Treatment = 55.5 (mean)	Control n = 134 (male) n = 93 (female) Treatment n = 151 (male) n = 124 (female)

### Definition of peer support

Of the articles included in this study (n=22), there were only six that defined peer support, and these definitions varied in their explanations. Three studies described peers as a group of individuals who have experienced a major limb amputation.^21–23^ Peer support involved individuals with amputation sharing their lived experiences, either one-on-one or between groups of individuals. ^21,22,24–26^ Observing peers further along in their amputation recovery was defined as a form of peer support.^21^ Three articles indicated that peer support was an opportunity for amputees to educate each other on how to engage in activities of daily living.^22,24,26^ This included guidance on improving mobility and functionality, such as walking on uneven surfaces, moving through crowds and navigating stairs.^25^ In addition, amputees were able to receive emotional and moral support from their peers by providing inspiration and encouragement.^22,26^

### Implementation of Peer Support

Twelve studies discussed the types of settings in which peer support may be implemented. Seven of these studies described programs implemented in community settings, including charities, organizations, and clinics.^21,22,24,27–30^ Four studies described programs facilitated in rehabilitation settings.^25^,^31^-^33^ While one study discussed peer support being implemented within a hospital and community setting.^34^

Fifteen studies described the method by which peer support programs are delivered, which varied between formal and informal delivery formats. Eight studies outlined structured delivery methods in which participants engaged in organized peer support sessions led by a peer volunteer, with or without prior training in delivering peer support. ^21,24,27–30,34,35^ Seven studies characterized informal delivery approaches, depicting them as a natural and chance encounter with another amputee, where the opportunity for knowledge and sharing of experiences could be exchanged.^24,25,31–34,36^

### Outcomes in Peer Support Interventions

The effect of using peer support as a study intervention was explored by two RCTs.^29,30^ Physical health outcomes were measured using the Short Musculoskeletal Function Assessment, Chronic Pain Grade Questionnaire,^29^ and Brief Pain Inventory,^30^ which allowed for the assessment of musculoskeletal function and pain, respectively. The studies also examined psychological well-being outcomes such as depression, affect, and self-efficacy, including: the Patient Health Questionnaire Depression Module,^29^ the Centre for Epidemiologic Studies Depression,^30^ Positive and Negative Affect Schedule,**^30^** Positive States of Mind,**^30^** and a Modified Self-Efficacy Scale.^30^ Social support outcomes were assessed using a multidimensional scale of perceived social support. Lastly, the studies examined quality of life as an outcome, and this was assessed using the World Health Organization Quality of Life Scale^29^ and Satisfaction with Life Scale.^30^

### Benefits of Peer Support

All 22 articles included in this scoping review discussed the physical or psychosocial benefits of the provision of peer support.

#### Improved physical functioning

Two RCTs^29,30^ reported on the impact of peer support on physical function. Wegener et al.^30^ reported that participants experienced a decrease in functional limitations six months following their participation in a peer support intervention. However, Turner et al.^29^ did not report an improvement; nevertheless, the authors noted possible explanations to be a lower physical baseline prior to study involvement as the participants were older adults.^29^

#### Enhanced psychological well-being

Four studies explored the benefits of psychological well-being; these included two studies that examined peer support as a secondary objective^32,37^ and two RCTs.^29,30^ Three studies reported that peer support involvement led to a reduction in symptoms of depression in their participants.^29,30,36^ While Liu et al.^31^ found that talking to peers provided an opportunity to alleviate emotional distress.

Nineteen reported on the perceived benefits of social support and connectedness. The majority of these studies discussed how peer support provided an opportunity for participants to share experiences and give advice to each other, which allowed them to build social connections and aided in their recovery transition.^20,22–28,33–35,37–41^

Studies have also reported that peer support groups facilitate the exchange of knowledge and education on how to engage in daily life with an amputation, which is perceived to be more beneficial than support from a clinician.^21,24,28,34,35,37,40^

Ten of these articles discussed how engagement in amputee peer support groups provided participants with feelings of optimism and hope for the future.^21–25,31,36,38^ Amorelli et al.^21^ reported that when participants observed other individuals with amputation engage in daily activities, they felt hopeful that they could still enjoy life like they once had. Likewise, Richardson et al.^22^ discussed how peer support helped participants see that meaningful engagement in life can continue even after limb loss.

Seven articles discussed how amputee peer support groups facilitated the building of friendships and communities beyond those of family and non-amputee friends.^22,24,31,36,39–41^

The relationships built while engaging in peer support groups were reported by three studies to have reduced the perceived social isolation participants felt after experiencing their amputation.^24,31,37^ Two articles also discussed how the relationships built in peer support groups provided individuals with amputation with emotional and social support.^33,39^

#### Community participation and engagement

The impact of peer support on re-engaging in daily activities post-amputation was discussed in two qualitative studies.^21,38^ Amorelli et al.^21^ studied peer support as a primary objective, and both studies reported that the advice and support received from peers inspired participants' engagement in the community and daily activities.^38^

#### Impact on self-management, self-efficacy and well-being

Four of the studies reported on the perceived benefit that peer support had an impact on self-management and well-being.^27,30,32,37^ Participants reported that this type of social support boosted their self-esteem, confidence,^27,32,37^ and self-efficacy.^30^ In addition, this type of support group led to increased autonomy and well-being. One study, by Richardson et al.^22^ examined the impact of peer support from the mentors' perspective and found that it gave them a sense of purpose and a feeling of usefulness. Additionally, Wegener et al.^30^ measured the effect of a peer support intervention on self-efficacy and found it led to an increase in participants.

The acceptance and adaptation to major limb loss were explored in two qualitative studies^22,31^ and one RCT.^29^ The study by Liu et al.^31^ reported that observing peers' success with limb loss helped participants put their experiences into perspective. Richardson et al.^22^ examined the impact of providing support as a peer mentor and found that this was a useful experience, as it not only increased well-being, but it aided the peer mentor in adjusting to their limb loss. The RCT conducted by Turner et al.^29^ measured satisfaction with life using the World Health Quality of Life Scale. The study found greater improvements in participants led by a licensed health professional paired with a peer support compared to the control participants that were provided with educational materials and no support from a professional or peer.^29^

### Barriers and Risks in the Provision of Peer Support

Six studies discussed the perceived barriers and risks to the provision of peer support.

#### Physical and Organizational-Level Barriers

One cross-sectional study exploring peer support as a primary objective discussed a physical barrier to peer support.^26^ Participants in this study reported that the geographical distance of peer support meetings was inaccessible and inconvenient to them.

Perceived organizational barriers were discussed in two qualitative studies.^22,26^ Nathan et al. ^26^ reported that the short duration and frequency of peer support meetings deterred participants from joining groups. While Richardson et al.^22^ reported that participants felt there was a lack of formal training for peer mentors facilitating peer support programs, which resulted in uncertainty about the role of peers and their reputability.

#### Individual Level Barriers

Three qualitative studies discussed barriers specific to the individual.^22,24,26^ Participant reluctance to join support groups stemmed from various factors, encompassing feelings of self-consciousness regarding involvement,^24^ apprehension about opening up to peers and displaying vulnerability,^26^ or a perception that the discussed topics did not directly address their individual needs.^26^ The peer mentors facilitating peer support groups also expressed feelings of doubt and uncertainty about whether their delivery of peer support was beneficial to their participants.^22^

Two qualitative studies reported on the perceived risks of the provision of peer support.^22,28^ Richardson et al.^22^ reported that peer support was physically and emotionally burdensome for peer mentors and that there was a lack of support for their well-being. Mortimer et al.^28^ reported that the information discussed between peer mentors and mentees during informal peer support interventions can be misleading and cause distress to participants.

#### Barriers to group dynamics

Barriers to peer support group dynamics were discussed in four qualitative studies.^24,26,35,37^ Participants report that peer support groups can be intimidating to engage in due to their “cliquey” environments^24,26^ and they can increase an individual's negative thoughts about their amputation.^35^ Poor leadership and a lack of commonality among group members have also been described as factors deterring amputees from wanting to engage in peer support groups.^26^ Three studies discussed that their peer support groups were male-dominated, making it difficult for women to connect with other female amputees, and reported a lack of support available for addressing female-specific needs.^26,35,37^

## DISCUSSION

This scoping review describes the nature of the existing literature on peer support for individuals with major limb loss. Based on our findings, there appears to be no clear consensus on a definition of peer support. The literature also lacked comprehensive exploration of the implementation of peer support interventions, with only a limited number of studies detailing the various settings and delivery methods employed. Studies that discussed the settings of peer support gatherings mainly described them within community-based locations, and methods of delivery varied between formal and informal formats. Most of the research included in this review provided evidence through participants' perceived experiences. There were only two RCTs that explored peer support as an intervention; they measured outcomes related to physical, social, psychological, and quality of life domains. The benefits of peer-support interventions were highlighted in all articles, which included physical or psychosocial benefits. Conversely, there were few articles examining the barriers to peer support, which included organizational, individual, and group dynamic barriers.

Limited studies defined peer support, but the prevailing agreement among these studies was that peer support involves the sharing of experiences among individuals with major limb loss.^21–24,26^ The lack of clarity in defining peer support for major limb loss resonates with similar observations across other healthcare contexts.^43,44^ The inconsistency in definitions might be attributed to the diverse nature of peer support interventions and the contextual variations in their implementation. Comparable challenges in defining peer support have been identified in studies focusing on peer support for mental health and chronic disease populations.^43,44^ This may be due to the more recent introduction of peer support as an integral part of the healthcare system. This discrepancy underscores the need for standardized terminology to facilitate effective communication and comprehension among researchers, practitioners, and participants.

Peer support groups were positively reported by all studies included in this scoping review. The perceived benefit with the greatest amount of evidence was social support and connectedness. This was achieved through sharing experiences and giving or receiving advice from other group members.^22^ In a previous integrated review, Reichmann et al.^45^ similarly identified that peer support interventions benefited psychosocial outcomes during rehabilitation. The unique value of peer support provides participants with the opportunity to obtain reassurance from others in a similar position as themselves.^34^ For instance, peers with major limb losses reported that they were able to provide others with feelings of optimism and hope for the future.^21^ Similar claims were reported in peer support studies for individuals with diabetes, stating that sharing experiences enabled participants to receive validation from others when expressing their frustrations and concerns about their diabetes management.^46^ Significant emphasis on knowledge exchange and education facilitated in peer support groups was also positively highlighted throughout the review.^21,24–28,34,35,37,40^ According to Wasilewski et al.^16^ this is attributed to participants finding educational information more informative and engaging when delivered by a peer, as they were able to resonate better due to shared experiences. In addition, peer support led to the building of friendships and a community for individuals with major limb loss.^20,22,24,31,39–41^ Peers were able to share advice amongst each other, which inspired them to engage more in the community, in daily activities, and build a life outside their family and non-amputee friends.^16,42,47^

In this review, findings suggest participants expressed concerns regarding inaccessibility (e.g., geographical distance) and the inconvenience of attending peer support meetings.^26,48^ Flexible communication options, like telephone or online platforms, have been proposed as solutions and proven effective in increasing attendance frequency and participation.^20,39,46^ A meta-analysis of patients with diabetes found that support groups offered through telephone-based communications were equally effective as in person.^16^ Additionally, barriers to participation or engagement occurred due to a lack of peer mentor training, which resulted in poor leadership skills and self-doubt among participants and mentors.^22,26^

The review underscores the potential benefits of peer support, particularly in terms of social support, connectedness, knowledge exchange, and community-building. However, research on the effectiveness of peer support interventions from RCTs is limited. Moreover, this research highlighted challenges to participation and engagement, such as accessibility and group dynamics. Future research should focus on developing and evaluating flexible communication options, like online platforms, to overcome geographical barriers. Additionally, comprehensive mentor training, mentor-mentee relationship-building, and tailored group categorization should be further explored to enhance engagement and address dynamics within peer support groups.

### Strengths and Limitations

The strength of this scoping review is in the methodological rigour of the approach. This review followed Arksey and O'Malley's methodological framework and was guided by a university-affiliated scientific librarian. As a result, this review provides a comprehensive summary of existing literature on the topic of peer support specific to individuals with amputations. This scoping review had some limitations. First, most of the articles included in this study did not explore peer support as their primary objective. This and the lack of RCTs made it difficult for the authors to understand the direct impact of peer support on health outcomes, as most studies only focused on its perceived benefits. This limited our study objective to understanding the impact of peer support as an intervention.

## CONCLUSION

This scoping review provided an understanding of what is known in the literature about peer support and people with major limb amputations. For this population, studies have shown that there are many perceived benefits to the provision of peer support. However, given the small number of studies in this field, future research is needed to explore the implementation process and evaluate the effectiveness of peer support for this population.

## DECLARATION OF CONFLICTING INTERESTS

The authors report that there are no conflicts of interest to declare.

## AUTHOR CONTRIBUTION

- **Annamaria Costa-Parke:** responsible for researching and reviewing all included articles and co-writing manuscript.
- **Anna Maria Di Lella:** responsible for researching and reviewing all included articles and co-writing manuscript.
- **Ashley Walker:** responsible for editing and co-writing manuscript.
- **Lee Verweel:** conceptualizing project, responsible for editing, co-writing manuscript, project supervision.
- **Crystal MacKay:** conceptualizing project, responsible for editing, co-writing manuscript, project supervision.

## SOURCES OF SUPPORT

No funding was provided for this review.

## APPENDICIES

### Legend

**Table:**

Operator	Meaning
**.tw**	title or abstract
**.kf**	word in author provided keyword
**.tw,kf**	the term is found in the title, abstract or in an author provided keyword
*** or $**	truncation (E.g., support OR supports OR supporting)
**or/#-#**	Refers to the lines that include words related to the search term (eg. or/1–10 [amputation] means that lines 1 or 2 or 3 or 4 or 5 or 6 or 7 or 8 or 9 or 10 include terms related to amputation)
**adj#**	the search term is within a specified number of words of the term (E.g. peer adj2 is within 2 words of the term support* (e.g., peer support OR supporting a peer))

## **APPENDIX A: Ovid MEDLINE** (Timeline: 1946 to 2023)

**Table:**

#	Searches	Results
1	amputation/or disarticulation/or hemipelvectomy/	23917
2	Amputation, Traumatic/or leg amputation/or above knee amputation/or below knee amputation/or knee amputation/or arm amputation/or amputation/or foot amputation/or hand amputation/or limb amputation/	27952
3	(Major limb amputat* or major limb loss or limb absence or limb loss).tw,kf.	2524
4	(Lower limb amputation* or Upper limb amputation*).tw,kf.	2801
5	Amputation Stumps/	3218
6	Amputation, Surgical/	182
7	Artificial Limbs.tw.kf.	587
8	amputat* stump*.tw,kf.	1095
9	Artificial Limbs/	7930
10	((leg or knee or arm or foot or hand or wrist or ankle or limb) adj3 amputat*).tw,kf.	12980
11	or/1–10 [Amputation]	42690
12	Social support/	78053
13	(peer adj2 (group* or support* or coach* or mentor*)).tw,kf.	13117
14	peer group/	23804
15	Mentoring/	3699
16	(social adj2 support).tw,kf.	54253
17	(Iay$ adj2 (led or run or help$ or support$ or visit$ or based or deliver$ or worker? or person$)).tw,kf.	8855
18	(user$ adj2 (led or run or help$ or support$ or visit$ or based or deliver$)).tw,kf.	7513
19	(voluntary adj2 (work$ or care or involvement or help$ or counsel$)).tw,kf.	2660
20	(volunteer adj2 (work$ or care or involvement or help$ or counsel$)).tw,kf.	1240
21	(community adj2 (person$ or based or visit$ or worker?)).tw,kf.	88377
22	(support adj2 (person$ or worker?)).tw,kf.	7020
23	(mutual adj2 (aid or support?)).tw,kf.	1512
24	(expert adj2 patient?).tw,kf.	1268
25	(non adj2 (professional? or medical)).tw,kf.	12426
26	(supportive adj2 relationship).tw,kf.	310
27	(health adj2 coach$).tw,kf.	1438
28	peer* to peer* support.tw.kf.	200
29	patient* to patient* support.tw,kf.	29
30	survivor* network*.tw,kf.	51
31	or/12–30 [Peer Support]	260846
32	11 and 31	300

## **Appendix B: EMBASE** (Timeline: 1947 to 2023)

**Table:**

#	Searches	Results
1	amputation/or disarticulation/or hemipelvectomy/	35000
2	Amputation, Traumatic/or leg amputation/or above knee amputation/or below knee amputation/or knee amputation/or arm amputation/or amputation/or foot amputation/or hand amputation/or limb amputation/	59817
3	(Major limb amputat* or major limb loss or limb absence or limb loss).tw,kf.	3357
4	(Lower limb amputation* or Upper limb amputation*).tw,kf.	3860
5	Amputation Stumps/	7003
6	Amputation, Surgical/	0
7	Artificial Limbs.tw,kf.	594
8	amputat* stump*.tw,kf.	1193
9	Artificial Limbs/	4445
10	((leg or knee or arm or foot or hand or wrist or ankle or limb) adj3 amputat*).tw,kf.	18335
11	or/1–10 [Amputation]	73823
12	Social support/	109599
13	(peer adj2 (group* or support* or coach* or mentor*)).tw,kf.	17481
14	peer group/	29351
15	Mentoring/	6087
16	(social adj2 support).tw,kf.	66633
17	(lay$ adj2 (led or run or help$ or support$ or visit$ or based or deliver$ or worker? or person$)).tw,kf.	9821
18	(user$ adj2 (led or run or help$ or support$ or visit$ or based or deliver$)).tw,kf.	9109
19	(voluntary adj2 (work$ or care or involvement or help$ or counsel$)).tw,kf.	3125
20	(volunteer adj2 (work$ or care or involvement or help$ or counsel$)).tw,kf.	1615
21	(community adj2 (person$ or based or visit$ or worker?)).tw,kf.	111367
22	(support adj2 (person$ or worker?)).tw,kf.	9212
23	(mutual adj2 (aid or support?)).tw,kf.	1877
24	(expert adj2 patient?).tw,kf.	2312
25	(non adj2 (professional? or medical)).tw,kf.	19072
26	(supportive adj2 relationship).tw,kf.	406
27	(health adj2 coach$).tw,kf.	2084
28	peer* to peer* support.tw,kf.	328
29	patient* to patient* support.tw,kf.	51
30	survivor* network*.tw,kf.	78
31	or/12–30 [Peer Support]	322841
32	11 and 31	514

## **Appendix C: PsycInfo** (Timeline: 1806 to 2023)

**Table:**

#	Searches	Results
1	amputation/or disarticulation/or hemipelvectomy/	1109
2	“Amputation, Traumatic”/or “leg amputation”/or “above knee amputation”/or “below knee amputation”/or “knee amputation”/or “arm amputation”/or amputation/or “foot amputation”/or “hand amputation”/or “limb amputation”/	1109
3	(“Major limb amputat*” or “major limb loss” or “limb absence” or “limb loss”).ti,ab,id.	154
4	(“Lower limb amputation*” or “Upper limb amputation*”).ti,ab,id.	251
5	“Amputation Stumps”/	0
6	“Amputation, Surgical”/	0
7	“Artificial Limbs”.ti,ab,id.	41
8	“amputat* stump*”.ti,ab,id.	22
9	“Artificial Limbs”/	1121
10	((leg or knee or arm or foot or hand or wrist or ankle or limb) adj3 amputat*).ti,ab,id.	762
11	1 or 2 or 3 or 4 or 5 or 6 or 7 or 8 or 9 or 10 [Amputation]	2387
12	“Social support”/	42141
13	(peer adj2 (group* or support* or coach* or mentor*)).ti,ab,id.	15696
14	“peer group”/	0
15	Mentoring/	0
16	(social adj2 support).ti,ab,id.	61395
17	(lay# adj2 (led or run or help# or support# or visit# or based or deliver# or worker? or person#)).ti,ab,id.	4
18	(user# adj2 (led or run or help# or support# or visit# or based or deliver#)).ti,ab,id.	850
19	(voluntary adj2 (work# or care or involvement or help# or counsel#)).ti,ab,id.	228
20	(volunteer adj2 (work# or care or involvement or help# or counsel#)).ti,ab,id.	208
21	(community adj2 (person# or based or visit# or worker?)).ti,ab,id.	38917
22	(support adj2 (person# or worker?)).ti,ab,id.	2600
23	(mutual adj2 (aid or support?)).ti,ab,id.	1911
24	(expert adj2 patient?).ti,ab,id.	260
25	(non adj2 (professional? or medical)).ti,ab,id.	4077
26	(supportive adj2 relationship).ti,ab,id.	528
27	(health adj2 coach#).ti,ab,id.	2
28	“peer* to peer* support”.ti,ab,id.	115
29	“patient* to patient* support”.ti,ab,id.	7
30	“survivor* network*”.ti,ab,id.	30
31	12 or 13 or 14 or 15 or 16 or 17 or 18 or 19 or 20 or 21 or 22 or 23 or 24 or 25 or 26 or 27 or 28 or 29 or “30 [Peer Support]”.mp. [mp=title, abstract, heading word, table of contents, key concepts, original title, tests & measures, mesh word]	132455
32	11 and 31	82

## **Appendix D: CINAHL** (Timeline: 1991 to 2023)

**Table:**

#	Query	Limiters/Expanders	Last Run Via	Results
S32	S11 AND S31	Search modes - Boolean/Phrase	Interface - EBSCOhost Research Databases Search Screen - Advanced Search Database - CINAHL	187
S31	S12 OR S13 OR S14 OR S15 OR S16 OR S17 OR S18 OR S19 OR S20 OR S21 OR S22 OR S23 OR S24 OR S25 OR S26 OR S27 OR S28 OR S29 OR S30	Search modes - Boolean/Phrase	Interface - EBSCOhost Research Databases Search Screen - Advanced Search Database - CINAHL	131,387
S30	(Tl “survivor* network*” OR AB “survivor* network*” OR SU “survivor* network*”)	Search modes - Boolean/Phrase	Interface - EBSCOhost Research Databases Search Screen - Advanced Search Database - CINAHL	34
S29	(Tl “patient* to patient* support” OR AB “patient* to patient* support” OR SU “patient* to patient* support”)	Search modes - Boolean/Phrase	Interface - EBSCOhost Research Databases Search Screen - Advanced Search Database - CINAHL	1
S28	(Tl “peer* to peer* support” OR AB “peer* to peer* support” OR SU “peer* to peer* support”)	Search modes - Boolean/Phrase	Interface - EBSCOhost Research Databases Search Screen - Advanced Search Database - CINAHL	106
S27	((Tl health OR AB health OR SU health) N2 (Tl coach? OR AB coach? OR SU coach?))	Search modes - Boolean/Phrase	Interface - EBSCOhost Research Databases Search Screen - Advanced Search Database - CINAHL	461
S26	((Tl supportive OR AB supportive OR SU supportive) N2 (Tl relationship OR AB relationship OR SU relationship))	Search modes - Boolean/Phrase	Interface - EBSCOhost Research Databases Search Screen - Advanced Search Database - CINAHL	1,276
S25	((Tl non OR AB non OR SU non) N2 ((Tl professional# OR AB professional# OR SU professional#) OR (Tl medical OR AB medical OR SU medical)))	Search modes - Boolean/Phrase	Interface - EBSCOhost Research Databases Search Screen - Advanced Search Database - CINAHL	6,442
S24	((Tl expert OR AB expert OR SU expert) N2 (Tl patient# OR AB patient# OR SU patient#))	Search modes - Boolean/Phrase	Interface - EBSCOhost Research Databases Search Screen - Advanced Search Database - CINAHL	1,843
S23	((Tl mutual OR AB mutual OR SU mutual) N2 ((Tl aid OR AB aid OR SU aid) OR (Tl support# OR AB support# OR SU support#)))	Search modes - Boolean/Phrase	Interface - EBSCOhost Research Databases Search Screen - Advanced Search Database - CINAHL	1,175
S22	((Tl support OR AB support OR SU support) N2 ((Tl person? OR AB person? OR SU person?) OR (Tl worker# OR AB worker# OR SU worker#)))	Search modes - Boolean/Phrase	Interface - EBSCOhost Research Databases Search Screen - Advanced Search Database - CINAHL	5,168
S21	((Tl community OR AB community OR SU community) N2 ((Tl person? OR AB person? OR SU person?) OR (Tl based OR AB based OR SU based) OR (Tl visit? OR AB visit? OR SU visit?) OR (Tl worker# OR AB worker# OR SU worker#)))	Search modes - Boolean/Phrase	Interface - EBSCOhost Research Databases Search Screen - Advanced Search Database - CINAHL	53,178
S20	((Tl volunteer OR AB volunteer OR SU volunteer) N2 ((Tl work? OR AB work? OR SU work?) OR (Tl care OR AB care OR SU care) OR (Tl involvement OR AB involvement OR SU involvement) OR (Tl help? OR AB help? OR SU help?) OR (Tl counsel? OR AB counsel? OR SU counsel?)))	Search modes - Boolean/Phrase	Interface - EBSCOhost Research Databases Search Screen - Advanced Search Database - CINAHL	1,411
S19	((Tl voluntary OR AB voluntary OR SU voluntary) N2 ((Tl work? OR AB work? OR SU work?) OR (Tl care OR AB care OR SU care) OR (Tl involvement OR AB involvement OR SU involvement) OR (Tl help? OR AB help? OR SU help?) OR (Tl counsel? OR AB counsel? OR SU counsel?)))	Search modes - Boolean/Phrase	Interface - EBSCOhost Research Databases Search Screen - Advanced Search Database - CINAHL	655
S18	((Tl user? OR AB user? OR SU user?) N2 ((Tl led OR AB led OR SU led) OR (Tl run OR AB run OR SU run) OR (Tl help? OR AB help? OR SU help?) OR (Tl support? OR AB support? OR SU support?) OR (Tl visit? OR AB visit? OR SU visit?) OR (Tl based OR AB based OR SU based) OR (Tl deliver? OR AB deliver? OR SU deliver?)))	Search modes - Boolean/Phrase	Interface - EBSCOhost Research Databases Search Screen - Advanced Search Database - CINAHL	3,951
S17	((Tl lay? OR AB lay? OR SU lay?) N2 ((Tl led OR AB led OR SU led) OR (Tl run OR AB run OR SU run) OR (Tl help? OR AB help? OR SU help?) OR (Tl support? OR AB support? OR SU support?) OR (Tl visit? OR AB visit? OR SU visit?) OR (Tl based OR AB based OR SU based) OR (Tl deliver? OR AB deliver? OR SU deliver?) OR (Tl worker# OR AB worker# OR SU worker#) OR (Tl person? OR AB person? OR SU person?)))	Search modes - Boolean/Phrase	Interface - EBSCOhost Research Databases Search Screen - Advanced Search Database - CINAHL	1,128
S16	((Tl social OR AB social OR SU social) N2 (Tl support OR AB support OR SU support))	Search modes - Boolean/Phrase	Interface - EBSCOhost Research Databases Search Screen - Advanced Search Database - CINAHL	38,938
S15	(MH Mentoring)	Search modes - Boolean/Phrase	Interface - EBSCOhost Research Databases Search Screen - Advanced Search Database - CINAHL	0
S14	(MH “peer group”)	Search modes - Boolean/Phrase	Interface - EBSCOhost Research Databases Search Screen - Advanced Search Database - CINAHL	15,928
S13	((Tl peer OR AB peer OR SU peer) N2 ((Tl group* OR AB group* OR SU group*) OR (Tl support* OR AB support* OR SU support*) OR (Tl coach* OR AB coach* OR SU coach*) OR (Tl mentor* OR AB mentor* OR SU mentor*)))	Search modes - Boolean/Phrase	Interface - EBSCOhost Research Databases Search Screen - Advanced Search Database - CINAHL	23,113
S12	(MH “Social support”)	Search modes - Boolean/Phrase	Interface - EBSCOhost Research Databases Search Screen - Advanced Search Database - CINAHL	0
S11	S1 OR S2 OR S3 OR S4 OR S5 OR S6 OR S7 OR S8 OR S9 ORS10	Search modes - Boolean/Phrase	Interface - EBSCOhost Research Databases Search Screen - Advanced Search Database - CINAHL	12,972
S10	(((Tl leg OR AB leg OR SU leg) OR (Tl knee OR AB knee OR SU knee) OR (Tl arm OR AB arm OR SU arm) OR (Tl foot OR AB foot OR SU foot) OR (Tl hand OR AB hand OR SU hand) OR (Tl wrist OR AB wrist OR SU wrist) OR (Tl ankle OR AB ankle OR SU ankle) OR (Tl limb OR AB limb OR SU limb)) N3 (Tl amputat* OR AB amputat* OR SU amputat*))	Search modes - Boolean/Phrase	Interface - EBSCOhost Research Databases Search Screen - Advanced Search Database - CINAHL	6,306
S9	(MH “Artificial Limbs”)	Search modes - Boolean/Phrase	Interface - EBSCOhost Research Databases Search Screen - Advanced Search Database - CINAHL	0
S8	(Tl “amputat* stump*” OR AB “amputat* stump*” OR SU “amputat* stump*”)	Search modes - Boolean/Phrase	Interface - EBSCOhost Research Databases Search Screen - Advanced Search Database - CINAHL	553
S7	(Tl “Artificial Limbs” OR AB “Artificial Limbs” OR SU “Artificial Limbs”)	Search modes - Boolean/Phrase	Interface - EBSCOhost Research Databases Search Screen - Advanced Search Database - CINAHL	56
S6	(MH “Amputation, Surgical”)	Search modes - Boolean/Phrase	Interface - EBSCOhost Research Databases Search Screen - Advanced Search Database - CINAHL	0
S5	(MH “Amputation Stumps”)	Search modes - Boolean/Phrase	Interface - EBSCOhost Research Databases Search Screen - Advanced Search Database - CINAHL	498
S4	((Tl “Lower limb amputation*” OR AB “Lower limb amputation*” OR SU “Lower limb amputation*”) OR (Tl “Upper limb amputation*” OR AB “Upper limb amputation*” OR SU “Upper limb amputation*”))	Search modes - Boolean/Phrase	Interface - EBSCOhost Research Databases Search Screen - Advanced Search Database - CINAHL	1,480
S3	((Tl “Major limb amputat*” OR AB “Major limb amputat*” OR SU “Major limb amputat*”) OR (Tl “major limb loss” OR AB “major limb loss” OR SU “major limb loss”) OR (Tl “limb absence” OR AB “limb absence” OR SU “limb absence”) OR (Tl “limb loss” OR AB “limb loss” OR SU “limb loss”))	Search modes - Boolean/Phrase	Interface - EBSCOhost Research Databases Search Screen - Advanced Search Database - CINAHL	1,010
S2	(MH “Amputation, Traumatic”) OR (MH “leg amputation”) OR (MH “above knee amputation”) OR (MH “below knee amputation”) OR (MH “knee amputation”) OR (MH “arm amputation”) OR (MH amputation) OR (MH “foot amputation”) OR (MH “hand amputation”) OR (MH “limb amputation”)	Search modes - Boolean/Phrase	Interface - EBSCOhost Research Databases Search Screen - Advanced Search Database - CINAHL	8,309
S1	(MH amputation) OR (MH disarticulation) OR (MH hemipelvectomy)	Search modes - Boolean/Phrase	Interface - EBSCOhost Research Databases Search Screen - Advanced Search Database - CINAHL	7,738
